# Case report: TP53 and RB1 loss may facilitate the transformation from lung adenocarcinoma to small cell lung cancer by expressing neuroendocrine markers

**DOI:** 10.3389/fendo.2022.1006480

**Published:** 2022-12-13

**Authors:** Jun Li, Bing Wei, Junnan Feng, Xinxin Wu, Yuxi Chang, Yi Wang, Xiuli Yang, Haiyan Zhang, Sile Han, Cuiyun Zhang, Jiawen Zheng, Harry J. M. Groen, Anke van den Berg, Jie Ma, Hongle Li, Yongjun Guo

**Affiliations:** ^1^ Department of Molecular Pathology, Clinical Pathology Center, Affiliated Cancer Hospital of Zhengzhou University and Henan Cancer Hospital, Zhengzhou, China; ^2^ Henan Key Laboratory of Molecular Pathology, Zhengzhou, China; ^3^ Henan International Joint Laboratory of Cancer Molecular Genetics, Zhengzhou, China; ^4^ Department of Pathology, Clinical Pathology Center, Affiliated Cancer Hospital of Zhengzhou University and Henan Cancer Hospital, Zhengzhou, China; ^5^ Department of Oncology, First Affiliated Hospital of Nanyang Medical College, Nanyang, China; ^6^ Department of Pathology, First Affiliated Hospital of Nanyang Medical College, Nanyang, China; ^7^ Department of Pulmonary Diseases, University of Groningen, University Medical Center Groningen, Groningen, Netherlands; ^8^ Department of Pathology and Medical Biology, University of Groningen, University Medical Center Groningen, Groningen, Netherlands

**Keywords:** small cell lung cancer transformation, lung adenocarcinoma, osimertinib, TP53 and RB1 loss, MYC amplification

## Abstract

**Introduction:**

Transformation from lung adenocarcinoma (LUAD) to small cell lung cancer (SCLC) is one of the mechanisms responsible for acquired EGFR-TKIs resistance. Although it rarely happens this event determines a rapid disease deterioration and needs specific treatment.

**Patient and method:**

We report a case of 75-year-old LUAD female with a p.L858R mutation in Epidermal Growth Factor Receptor (EGFR) who presented with SCLC transformation after responding to first line osimertinib treatment for only 6 months. To understand the underlying molecular mechanism, we retrospectively sequenced the first (LUAD) and the second (SCLC) biopsy using a 56 multi-gene panel. Immunohistochemistry (IHC) staining and Fluorescence In Situ Hybridization (FISH) was applied to confirm the genetic aberrations identified.

**Results:**

EGFR p.E709A and p.L858R, Tumor Protein p53 (TP53) p.A159D and Retinoblastoma 1 (RB1) c.365-1G>A were detected in both the diagnostic LUAD and transformed SCLC samples. A high copy number gain for Proto-Oncogene C-Myc (MYC) and a Phosphoinositide 3-Kinase Alpha (PIK3CA) p.E545K mutation were found in the transformed sample specifically. Strong TP53 staining and negative RB1 staining were observed in both LUAD and SCLC samples, but FISH only identified MYC amplification in SCLC tissue.

**Conclusion:**

We consider the combined presence of MYC amplification with mutations in TP53 and RB1 as drivers of SCLC transformation. Our results highlight the need to systematically evaluate TP53 and RB1 status in LUAD patients to offer a different therapeutic strategy.

## Introduction

Transformation from *EGFR* mutant LUAD to SCLC is one of the mechanisms underlying the acquired resistance to EGFR tyrosine kinase inhibitors (TKIs) (1). The reported frequency of SCLC transformation ranges from 5% to 14% (2, 3), but the disease usually becomes highly aggressive when it occurs (4). Although the precursors of transformed SCLC cells are still inconclusive, it would be of great value to find markers that can predict the transformation risk efficiently. This is because the treatment strategies for SCLC are substantially different from LUAD (5). For limited stage disease, concurrent chemoradiation is usually used for SCLC treatment but surgery is rarely recommended; for LUAD, radical surgery is still the mainstay of treatment, although other adjuvant treatments, *i.e.*, radiotherapy, chemotherapy and immunotherapy either given alone or in combination, have shown promising clinical outcomes. For advanced disease, chemotherapy is the mainstay of treatment for both LUAD and SCLC but with different regimens. Here, we report an LUAD case suffered from SCLC transformation with the treatment of osimertinib for only 6 months. Mutation screening was performed on the FFPE tissues from both primary LUAD and transformed SCLC malignancies to understand its evolution.

## Case presentation

**Figure 1:**
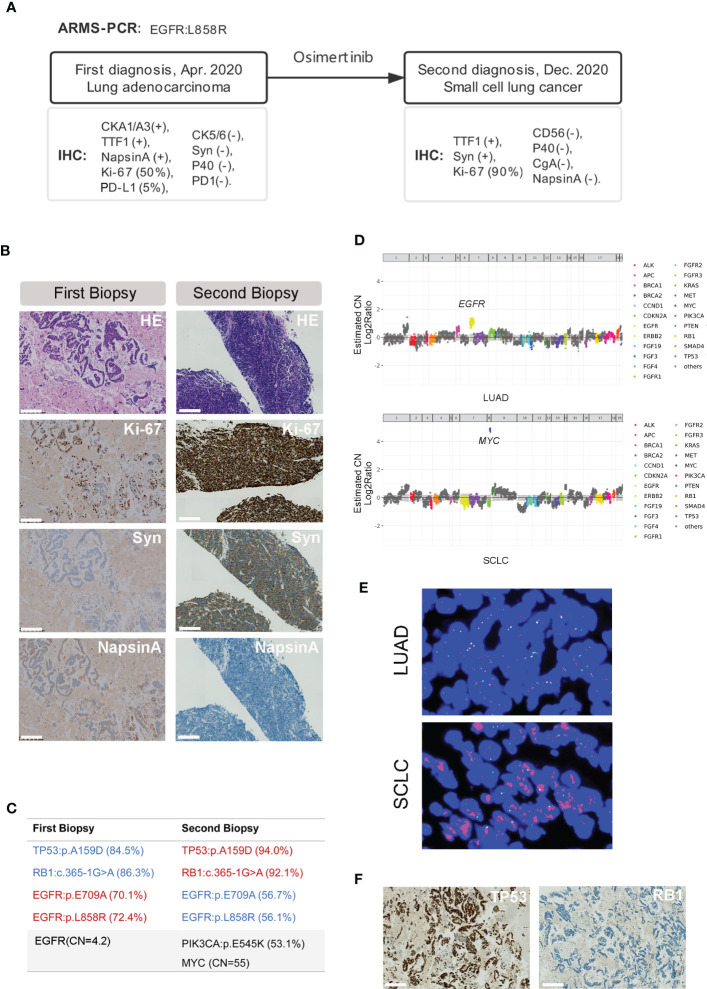
Pathology features and genetic aberrations present before and after SCLC transformation. **(A)** A brief history of the disease diagnosis and treatment in this patient. **(B)** HE and IHC staining of the tumor tissue before (first biopsy) and after (second biopsy) the SCLC transformation (10×). Scale bar indicates 200μm. **(C)** Genetic aberrations detected in the first and second tumor biopsy tissue using the targeted sequencing covering 56 lung cancer related genes. **(D)** *MYC* copy number (CN=55, upper) and *EGFR* copy number (CN=4.2) determined by modeling copy number variation and aneuploidy across the genome, the Y axis is indicated as log2 scale. **(E)** Example of *MYC* amplification detected by FISH in the primary LUAD (upper) and transformed SCLC (lower) tissue (40×). **(F)** IHC staining pattern of TP53 and RB-1 in the LUAD biopsy before transformation (10×). Scale bar indicates 200μm.

A 75-year-old non-smoking female presented with chronic cough and fatigue as her main complaint and was admitted for medical tests and treatment to the hospital in April 2020. Physical examination was good with a performance score of one. Computed tomography (CT) scanning of the chest revealed a 52×43mm mass with several small nodules in the right lower lobe, and positron emission tomography (PET)-CT confirmed the hypermetabolic status of the nodules. Metastatic lesions were observed at the 9^th^ thoracic vertebrae and right 9^th^ rib bone. Tumor tissue of the main tumor mass was collected by fine needle aspiration (FNA) and processed into a formalin-fixed, paraffin-embedded (FFPE) block for histopathology diagnosis and molecular testing. Hematoxylin-eosin (HE) staining revealed a non-small cell lung cancer, with tumor cells staining positive for cytokeratin AE1/AE3 (CK AE1/AE3), thyroid transcription factor 1 (TTF-1) and Napsin A, and negative for cytokeratin 5/6 (CK5/6), synaptophysin (Syn) and P40, supporting a diagnosis of lung adenocarcinoma. Ki-67 and programmed death-ligand 1 (PD-L1) expression were found in about 50% and 5% of the tumor cells respectively. Programmed cell death protein 1 (PD1) was negative. An amplification-refractory mutation system (ARMS) test identified the common mutation of NM_005228.5(*EGFR*): c.2573T>G (p.Leu858Arg) (*EGFR* p.L858R) with an allele frequency of 72.4% in the tumor (ACCB Biotech Ltd, China; detection limit, 0.1%). The patient received oral osimertinib at a once-daily dose of 80mg as the first-line treatment. Follow-up CT imaging revealed a partial tumor response that continued for 6 months. Thereafter the patient developed disease progression. Hematoxylin and Eosin (HE) staining of the re-biopsy tumor tissue showed a small round morphology of the cells with high nuclear/cytoplasmic ratio and positive staining of Syn and TTF-1, and no staining for CD56, Chromogranin A (CgA), Napsin A and P40. The percentage of Ki-67 expression positive cells increased to about 90% (**Figures 1A, B**). Targeted capture sequencing of 56 lung cancer related genes (Burning Rock Dx, Guangzhou China) revealed following mutations: *EGFR* p.L858R (56.7%) and NM_005228.5(*EGFR*):c.2126A>C (p.Glu709Ala) (*EGFR* p.E709A, 56.7%), NM_006218.4(*PIK3CA*):c.1633G>A (p.Glu545Lys) (*PIK3CA* p.E545K, 53.1%), NM_000321.2 (*RB1*): c.365-1G>A (*RB1* c.365-1G>A, 92.1%) and NM_000546.6(*TP53*):c.476C>A (p.Ala159Asp) (*TP53* p.A159D, 93.98%), as well as copy number changes in *MYC* (CN=55). With this knowledge we also performed targeted capture sequencing analysis on the first tumor biopsy tissue. This revealed a similar mutational profile in the diagnostic tissue sample with the presence of *RB1* c.365-1G>A and the *TP53* p.A159D in addition to the *EGFR* p.L858R and p.E709A.Amplification in *EGFR* (CN=4.2), instead of *MYC*, was observed in the diagnostic LUAD sample (**Figures 1C, D**). Subsequent fluorescence *in situ* hybridization (FISH) confirmed the *MYC* amplification in the SCLC sample, while the diagnostic sample did not have the *MYC* amplification (**Figure 1E**). Additional immunohistochemistry revealed a positive staining for TP53^MUT^ (MAB-0674, Maixin Biotech., Fuzhou China) and negative staining for RB1 (MAB-0186, Maixin Biotech., Fuzhou China) on both the LUAD and transformed SCLC samples, consistent with the loss of function mutations caused by the *TP53* p.A159D and the *RB1* c.365-1G>A which leads to loss of a splice site (**Figure 1F**; **Supplementary Figure 1**).

Then the patient was treated with cisplatin plus irinotecan hydrochloride, but unfortunately passed away 4 months later. Collectively, this case showed a transformation from *EGFR* mutant adenocarcinoma to an aggressive neuroendocrine small-cell lung cancer (SCLC) after first-line osimertinib treatment in 6 months.

## Discussion

Functional loss of RB1 and TP53 are recurrent aberrations in transformed SCLC tumors, suggesting these aberrations might be highly predictive markers (1). However, TP53 and RB1 staining are not included in routine clinical care. Based on the updated CSCO and NCCN guidelines for non-small cell lung cancer, targeted sequencing panels usually only cover a subset of oncogenic driver genes, *i.e.*, *EGFR*, *Kirsten rat sarcoma virus* (*KRAS*), *Anaplastic lymphoma kinase* (*ALK*), *ROS Proto-Oncogene 1, Receptor Tyrosine Kinase* (*ROS1*), *B-Raf Proto-Oncogene, Serine/Threonine Kinase* (*BRAF*), *MET Proto-Oncogene, Receptor Tyrosine Kinase* (*MET*) and *Neurotrophin receptor tyrosine kinase* (*NTRK*), and do not provide information on *TP53* and *RB1*.

By targeted sequencing of the primary tumor tissue, we found both the *RB1* and *TP53* mutations were already present before start of osimertinib treatment. The *EGFR* p.L858R and p.E709A mutations were detected at comparable variant allele frequencies (VAFs) in both tumor samples, indicating that the mutations most likely occurred in cis. Although this specific combination of *EGFR* mutations is rare, mutations at E709 in *EGFR* usually present concurrently with other oncogenic *EGFR* mutations (6). Proliferation assay and colony formation assays revealed an oncogenic role of E790X in Ba/F3 and NIH-3T3 cells and dose-response experiments showed that E709K mutant cells were more sensitive to second-generation EGFR-TKIs than first- and third-generation drugs (7). Clinical data showed lower response rates of about 50% in patients with combined *EGFR* mutations including a p.E709X to first-generation EGFR-TKIs (8).

As compared to the primary LUAD tissue, the VAFs of *EGFR* p.L858R and p.E709A were decreased in the transformed SCLC tissue, and EGFR amplification was disappeared as well, indicating Osimertinib indeed inhibited the proliferation of the *EGFR*-mutant clone. In contrast, the VAFs of both *TP53* p.A159D and *RB1* c.365-1G>A increased, suggesting the transformed cells arise from a different clone. This observation is consistent with a series of cases have been reported that the original *EGFR* mutation could always be detected in the transformed SCLC tissues without exception (9–11). Unfortunately, the TP53 and RB1 status was unknown in these studies.

In recent years, several important studies pinpointed the essential role of TP53 and RB1 loss in the transformation from LUAD to SCLC. Offin et al. identified 43 out of 4112 lung cancer patients with concurrent *EGFR/TP53/RB1* mutations in their primary tumors, of which 7 were suffered from SCLC transformation after EGFR-TKIs treatment, 4 patients had small cell histology at the initial diagnosis, and the other 32 never had SCLC transformation till the last review (12). Of the patients with concurrent *EGFR/RB1* mutations (n=54), 11 patients didn’t have *TP53* mutation and none of them had SCLC transformation. In a sub-group of 142 cancer patients with *EGFR* mutation but not *TP53* and *RB1*, no transformation was observed. These results clearly showed *EGFR/RB1/TP53*-mutant patients had a significantly increased risk of SCLC transformation than the patients with mutations in one or two of these genes. In addition, *EGFR/RB1/TP53*-mutant patients showed worse clinical outcomes in both time to treatment discontinuation (TTD) and overall survival, as compared to the patients with only *EGFR/TP53* or *EGFR/RB1* mutations, indicating concurrent loss of RB1 and TP53 substantially changed the disease behavior. Actually, neither *EGFR* mutation, nor EGFR-TKIs treatment, is indispensable, since SCLC transformation were also observed in *EGFR* wild-type patients after immunotherapy, in *anaplastic lymphoma kinase* (*ALK*)-mutant patients after ALK-inhibitor treatment or immunotherapy (4).

Both *TP53* and *RB1* are well established tumor suppressor genes and mutated in a wide spectrum of cancers. A recent study investigating concurrent *RB1/TP53* mutations in pan-cancer revealed that *RB1* and *TP53* co-mutations were significantly enriched in small cell carcinomas and neuroendocrine carcinomas (13). Studies in animal models have shed light on understanding the biological context of SCLC transformation after TP53 and RB1 loss. Different from TP53, RB1 appears to play a more flexible role in regulation lineage-specific cell differentiation, evidenced by an increased number of lung neuroendocrine cells in *Rb1* conditional knockout mice during development (14). In addition, neuroendocrine markers are expressed in 39 out of 42 *RB1*-mutant SCLC cell lines, but are absence in 5/6 *RB1* wild-type SCLC cell lines. In prostate cancer mouse models, Ku et al. showed Rb1 loss facilitated the lineage plasticity of prostate cancer then additional Tp53 inactivation enabled the cells insensitive to antiandrogen therapy (15). However, it’s noteworthy that some of the *TP53/RB1*-mutant LUAD patients never transformed into SCLC, suggesting other factors are also involved (12).

In this case, we identified a *PIK3CA* p.E545K and a *MYC* amplification (CN=55) to be specific for the transformed SCLC tissue, indicating these aberrations may contribute to disease progression. The E545K is one of the hotspot mutations in *PIK3CA*, presenting in about 2% of NSCLC patients. This mutation affects the helical binding domain of PIK3CA. Different from the classic oncogenic mutations, *i.e.*, *EGFR* and *KRAS*, which are often mutually exclusive, *PIK3CA* mutations usually co-occur with other genetic aberrations in *EGFR*, *KRAS* or *ALK etc.* (16). Of note, the *PIK3CA* p.E545K is commonly observed in *EGFR* p.L858R mutant Chinese NSCLC patients (17). In a functional study, this mutation was shown to be sufficient to abrogate EGFR-TKI induced cellular apoptosis (18). Therefore, the occurrence of the *PIK3CA* p.E545K mutation might have attenuated the efficacy of osimertinib in this patient. Similar observations have been reported in other studies (19, 20). Although its role in the carcinogenesis of lung cancer is still controversial, it has been shown to promote the disease development in gallbladder and cervical cancer (21, 22).

MYC regulates a wide spectrum of biological processes involved in the initiation and progression of many cancers, and amplification of *MYC* has been observed in both lung adenocarcinoma and SCLC patients (23). *MYC* amplification has been associated with unfavorable clinical outcome and treatment resistance (24, 25). In SCLC, *MYC* amplification promotes cellular proliferation and inhibits cellular differentiation (26). We identified *MYC* amplification (CN=55) in the transformed SCLC tissue and not in the primary tumor. Besides *MYC* amplification, the SCLC sample also showed increased percentage of Ki-67 positive cells consistent with its role in proliferation. Of note. *MYC* was shown to promote rapid disease deterioration in *Tp53/Rb1* null SCLC mice and induced dynamic evolution in NEUROD1-expressing neuroendocrine-low SCLC subtype (24, 27). Taken together, these studies suggest that SCLC transformation and rapid disease deterioration as observed in this case might be contributed by the *TP53*/*RB1*-mutant profile in combination with the amplification of *MYC*. Although previous studies suggest that SCLC transformation is determined by multiple factors beyond genetic changes, *i.e.*, the origin and plasticity of the tumor cells (24, 27), *TP53* and *RB1* loss might be the key drivers in this case.

Considering the rapid disease progression and poor prognosis of patients with transformed SCLC, a lesson we learned from this case is the necessity to evaluate TP53 and RB1 status in *EGFR* mutated lung adenocarcinoma patients before start of EGFR-TKI treatment. This can be achieved by a high-throughput NGS test, or by IHC staining, which is more cost-effective. A critical question remains whether with the information currently available, we should treat these patients with chemotherapy in the first line or directly start with a combination of chemotherapy and osimertinib.

## Data availability statement

The original contributions presented in the study are included in the article/**Supplementary Materials**. Further inquiries can be directed to the corresponding authors.

## Ethics statement

The studies involving human participants were reviewed and approved by the ethics committee of Henan Cancer Hospital. The patients/participants provided their written informed consent to participate in this study. Written, informed consent was obtained from the participant for the publication of this case report (including all data and images).

## Author contributions

YG and HL conceived and designed the study, JL and BW analysed the data and drafted the manuscript with the support from HG and AB, JF, CZ and JZ performed the sequencing and bioinformatic analysis, SH, YW, YC and JM performed the IHC staining, FISH test and analysed the result, XY and HZ collected the clinical data. All authors contributed to the article and approved the submitted version.
