# Supplementary material for: Case report: TP53 and RB1 loss may facilitate the transformation from lung adenocarcinoma to small cell lung cancer by expressing neuroendocrine markers
Source: Front Endocrinol (Lausanne). 2022 Dec 13;13:1006480. doi: 10.3389/fendo.2022.1006480 (PMC9792468; doi:10.3389/fendo.2022.1006480)
Supplement: Supplementary file 1 [file Image_1.pdf]

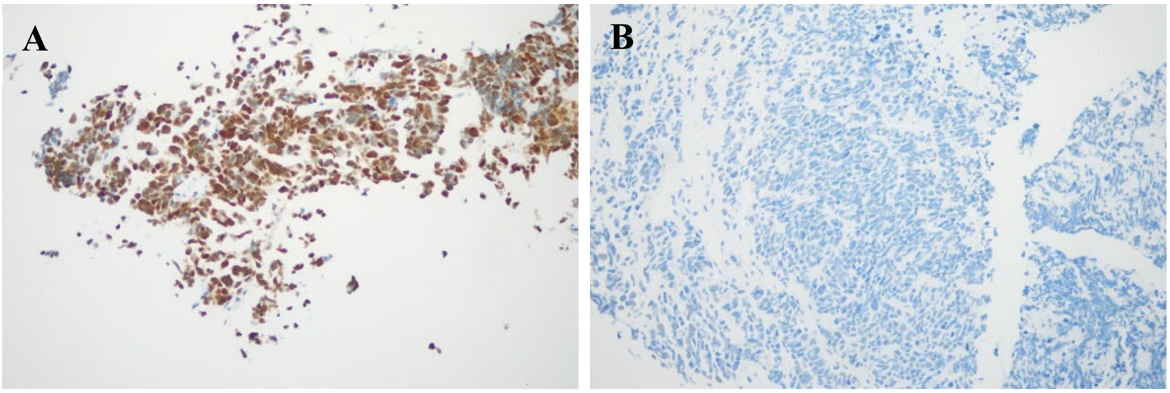

Supplementary Figure 1. Immunohistochemistry staining of TP53 and RB1 in the transformed SCLC tissue. IHC staining pattern of TP53 (A) and RB-1 (B) in the transformed SCLC biopsy (20×).
